# Effects of traditional Chinese exercises on the rehabilitation of patients with chronic heart failure: A meta-analysis

**DOI:** 10.3389/fpubh.2023.1139483

**Published:** 2023-02-23

**Authors:** Mengqiao Dai, Ziyan Luo, Shuqin Hu, Hu Chen, Jiechao Yang, Dandan Geng, Weina Li, Xiaoqin Liao

**Affiliations:** School of Nursing, Shanghai University of Traditional Chinese Medicine, Shanghai, China

**Keywords:** traditional Chinese exercises, Tai Chi, chronic heart failure, rehabilitation, meta

## Abstract

**Background:**

With the development of rehabilitation medicine, exercise therapy has gradually become one of the methods to prevent and treat cardiovascular diseases. It is widely used in clinic because it can further reduce the mortality rate, improve clinical symptoms, restore the activity ability of the body, improve the quality of life of patients and reduce the hospitalization rate. Traditional Chinese exercises have developed rapidly in recent years, which mainly include Baduanjin, Tai Ji, etc. However, meta-analyses of all types of exercises are not well characterized.

**Objectives:**

To evaluate the effect of traditional Chinese exercises (TCEs) on the rehabilitation of patients with chronic heart failure (CHF) using a meta-analysis.

**Methods:**

A systematic search of randomized controlled trials (RCTs) on TCEs for patients with CHF in 13 databases (PubMed, China National Knowledge Infrastructure, etc.). Meta-analysis was performed using Review Manager software (version 5.3) after two investigators independently screened the studies, assessed the quality of the studies, and extracted the data.

**Results:**

Meta-analysis of 21 randomized controlled trials which involved 1,665 patients with chronic heart failure showed that practicing TCEs was effective in improving patients' physiological outcomes such as VO_2_max [MD = 2.14, 95% CI (1.02, 3.26), *P* < 0.001], AT [MD = 1.61, 95% CI (1.06, 2.16), *P* < 0.001], and left ventricular ejection fraction [MD = 2.60, 95% CI (1.17, 4.02), *P* < 0.001]. Non-physiological outcomes benefited from the application of TCEs: 6-min walking distance [MD = 38.55, 95% CI (36.67, 40.42), *P* < 0.001], quality of life [MD = 5.52, 95% CI (3.17, 7.88), *P* < 0.001], and single-item TCM symptom scores in CHF patients: tiredness and fatigue [MD = 0.78, 95% CI (0.03, 1.53), *P* = 0.04], shortness of breath [MD = 0.44,95% CI (0.26, 0.62), *P* < 0.0001], facial puffiness and limb swelling [MD = 0.44,95% CI (0.12, 0.76), *P* = 0.007], palpitations [MD = 0.68,95% CI (0.14, 1.21), *P* = 0.01] were improved.

**Conclusions:**

TCEs improved several recovery indicators, heart failure-related clinical symptoms, quality of life, and physiological indicators in patients with CHF. It is worthwhile to expand the participants for practical application in clinical practice, but the existing evidence is insufficient and the heterogeneity of outcome is large. Therefore, more high-quality clinical trials are needed to support these results.

**Systematic review registration:**

PROSPERO, identifier [CRD42022383246].

## 1. Introduction

Chronic heart failure (CHF) is the end stage of many cardiovascular diseases. It is a complex syndrome that causes high hospitalization and mortality rates and places a significant burden on the public health system (1, 2). Studies show (3) that the absolute number of people with heart failure has nearly doubled since 1990. With the development of modern medicine, exercise rehabilitation has become one of the most important aspects of cardiac rehabilitation as one of the core elements of cardiac rehabilitation, enabling patients to improve their disease status through active exercise (4). The relevant guidelines (5)^1^ strongly recommend cardiac rehabilitation.

Traditional Chinese exercises (TCEs) combine internal and external training, rigidity, and flexibility, including Ba Duan Jin, Six-character formula, Taijiquan, Five mimic-animal exercises, and Zhan Zhuang. China's traditional exercises are based on traditional Chinese medicine, Yin and Yang, the theory of five elements, the science of channels and collaterals and the theory of Zang and fu, and China's traditional philosophic thinking and regimen concept are the upper concepts (6, 7). TCEs emphasize the harmony of the body and mind, and promote the operation of one's qi, blood, and fluids. After thousands of years of exploration and renewal, TCEs have evolved into the core of the idea of “adjusting the body, breath and mind,” through conscious inhaling and exhaling, relaxing body and mind, concentrating the mind to achieve the effect of disease prevention and cure, and consequently prolonging life. The good implementation effect of traditional Chinese exercises has been verified by meta-analysis in patients with hypertension (8), stroke (9), sleep disorder (10) and other chronic diseases. Practicing traditional Chinese exercises, for example, can effectively improve the gait of stroke patients, the pain symptoms of patients with chronic low back pain, the mood and symptoms of patients with sleep disorders etc. Studies have shown that traditional Chinese exercises as a sort of rhythmic and moderate-intensity aerobic exercises can promote physical ability, quality of life and health level (8). But the existing research on the rehabilitation effect of patients with chronic heart failure is lacking.

TCEs, as a non-pharmacological therapy with great cultural implications in China, have been used in the rehabilitation of patients with CHF because of their advantages of safety, economy, and not being restricted by venue and time (11). There are few studies on the intervention of chronic heart failure patients by traditional methods such as Baduanjin, Yijinjing, and Wuqinxi in China, and their universality and effectiveness need to be further tested. At present, there is a lack of a clear understanding of the regulatory variables (content, cycle, etc.) that affect the intervention effect of China's traditional exercises on patients with chronic heart failure.

However, the results of these clinical studies have varied widely. Some studies have shown that TCEs can effectively improve left ventricular ejection fraction (LVEF) (12–22), 6-min walking distance (6MWD) (13, 14, 16–19, 21–25), quality of life (QOL) (14–16), and other outcomes. Some related studies have shown that TCEs did not effectively affect patients' LVEF (15), 6WMD (17), and QOL (18, 19) compared with conventional medication or care, which was not statistically significant. However, caregivers need systematic scientific advice and guidelines to support them in developing relevant plans for their patients. Systematic evaluation of the effect of TCEs on the rehabilitation of patients with CHF is lacking.

At present, there is no best practice plan about different traditional Chinese exercises for the rehabilitation of CHF patients. This study evaluated the effect of TCEs on the rehabilitation of patients with CHF through meta-analysis to provide a reliable basis for clinical practice. This study also fills the gap in the current meta-analysis of related topics and provides an in-depth design idea for related clinical trials in the future. At the same time provides an effective practical path for the implementation of the “Key Project Planning for the Inheritance and Development of Chinese Excellent Traditional Culture.”

## 2. Methods

### 2.1. Search strategy

Randomized controlled trials (RCTs) on TCEs for patients with CHF were searched using a computerized retrieval system in the Chinese full-text journal database, VIP database, Wan Fang database, China National Knowledge Infrastructure (CNKI), WeiPu, WanFang, China Biology Medicine disc, National Medical Journal of China, PubMed, Cochrane Library, Web of Science, EBSCO, and Embase databases from the time of database construction to 25 November 2022. Chinese database mainly uses keywords such as “Ba Duan Jin,” “Taijiquan,” “Five mimic-animal exercises,” “Six-character formula,” “Qigong,” and “Yi Jin Jing,” “Five Elements Palm,” “Zhan Zhuang,” “Hui Chun Gong,” “Heart Failure,” “Chronic Heart Failure,” “cardiac insufficiency” for searching, and the specific search strategy is presented in Appendix. Thus, the search for the retrieval strategy for non-Chinese databases could be finalized (the concrete retrieval formula of the database is shown in Appendix). The reference lists of the relevant articles were screened and checked to identify more eligible studies.

### 2.2. Study inclusion and exclusion criteria

The inclusion criteria for studies were applied as follows. Study's participants: (1) Patients aged ≥18 years. (2) Recognized or authoritative guideline criteria confirmed the diagnosis of CHF. (3) Patients with NYHA cardiac function class I to III. (4) The patient does not have other serious complications. (5) The patient does not have physical activity disorder or cognitive impairment. Interventions: (1) Patients in the experimental group were given a single traditional Chinese exercise (e.g., Ba Duan Jin, Yi Jin Jing, Five mimic-animal exercises, Six-character formula, etc.). Study design: randomized controlled trial (RCT). Study language: Chinese or English.

The exclusion criteria for studies were applied as follows. (1) The intervention did not match or combined with the other instruments. (2) The intervention participants do not meet the inclusion criteria or is not clear. (3) Book or conference study. (4) Unavailability of the complete study.

### 2.3. Data extraction and quality assessment

The title and abstract of each retrieved study were read by two independent reviewers (MengQiao Dai and ZiYan Luo) who had undergone an evidence-based nursing course. The study was initially screened manually, while it was imported into EndNoteX9 for duplication. The remaining studies were again read in their entirety, and those that did not meet the criteria were removed according to the exclusion criteria. Two reviewers independently extracted and checked the information about the study, including author characteristics, year of publication, participants, type of study design, specific measures of the control and intervention groups, duration of the intervention, outcomes (LVEF, VO_2_max, AT, QOL, 6MWD, single-item TCM symptom scores), and the evaluation tool used for the outcome indicator. In case of disagreements between the two reviewers in the above steps, a third reviewer with the same qualifications was asked to discuss the decision.

Two reviewers performed an independent quality assessment of all the included studies. The Cochrane 5.1.0 quality evaluation criteria were used which consisted of seven items, and the reviewers made “low risk,” “high risk,” and “unclear” classifications for each item. If a study fully satisfied the criteria, the study was considered to have a low possibility of bias i.e., was classified as grade A; partially satisfied as grade B, the study was considered to have a moderate possibility of bias; completely unsatisfied as grade C; the study was considered to have a high possibility of bias.

### 2.4. Statistical analyses

Meta-analysis was performed using RevMan 5.3 software, and the count data were expressed as relative risk (RR) with 95% CI; continuous variables, such as maximal oxygen uptake (VO_2_max), anaerobic threshold (AT), LVEF, 6WMD, QOL, and traditional Chinese medicine (TCM) evidence alone scores, were expressed as mean difference (MD) or standardized mean difference (SMD) with 95% CI. If *P* > 0.05, *I*^2^ < 50%, there was no significant statistical heterogeneity among the studies, and a meta-analysis was performed using a fixed-effects model. If *P* < 0.05, *I*^2^ > 50%, there was greater heterogeneity among the studies, and a random-effects model was used to calculate the combined effect size. A subgroup analysis was conducted to further explore the sources of heterogeneity. Statistical significance was set at *P* < 0.05.

## 3. Results

### 3.1. Results of literature retrieval

**Figure 1:**
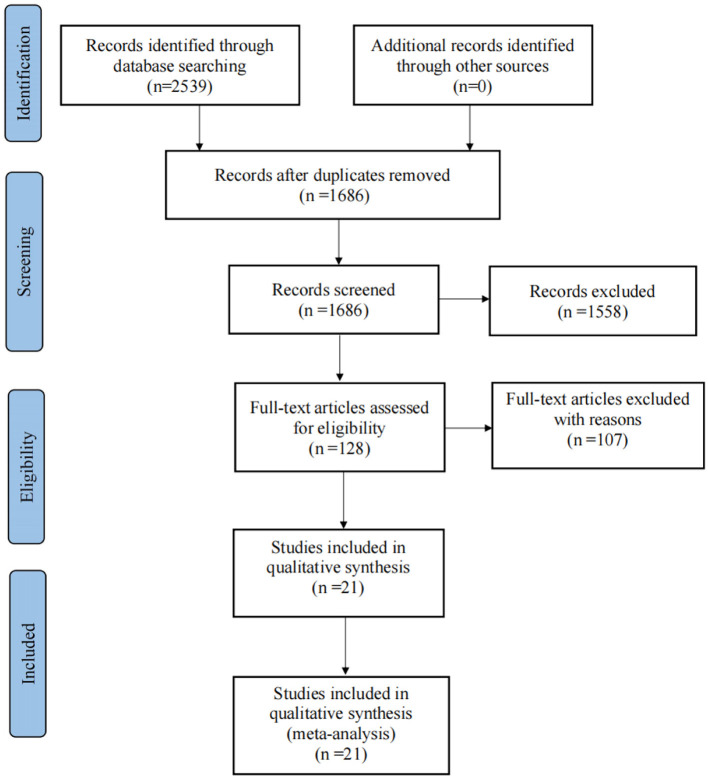
Flow diagram of search and selection of studies.

A total of 2,539 documents were obtained by computer and manual search, including 1,827 documents in English and 712 documents in Chinese, and a total of 853 duplicate documents were removed using Endnote software manually. After reading the titles and abstracts of the remaining studies, 1,558 irrelevant pieces of study were removed. The full text of the remaining 128 papers was read according to the inclusion and exclusion criteria of the study, and 21 papers were finally included in the final statistical analysis. A flow diagram of the search and selection of the studies is shown in Figure 1.

### 3.2. Study characteristics

The 21 included papers (12–32) were sorted by article. A total of 1,665 patients with CHF were included. In this study, the sample size ranged from 18 to 150. The time span for literature publication was from 2016 to 2022. Among them, 17 were Chinese studies and four were English studies. The basic characteristics of the included studies are presented in Supplementary Table 1.

### 3.3. Risk of bias assessment

**Figure 2:**
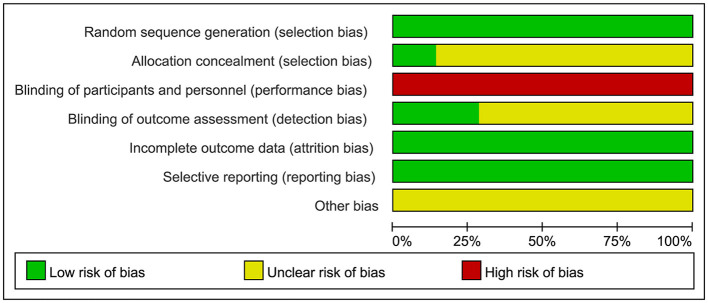
Bias risk diagram: judgment of risk of bias expressed as a percentage of all included studies.

**Figure 3:**
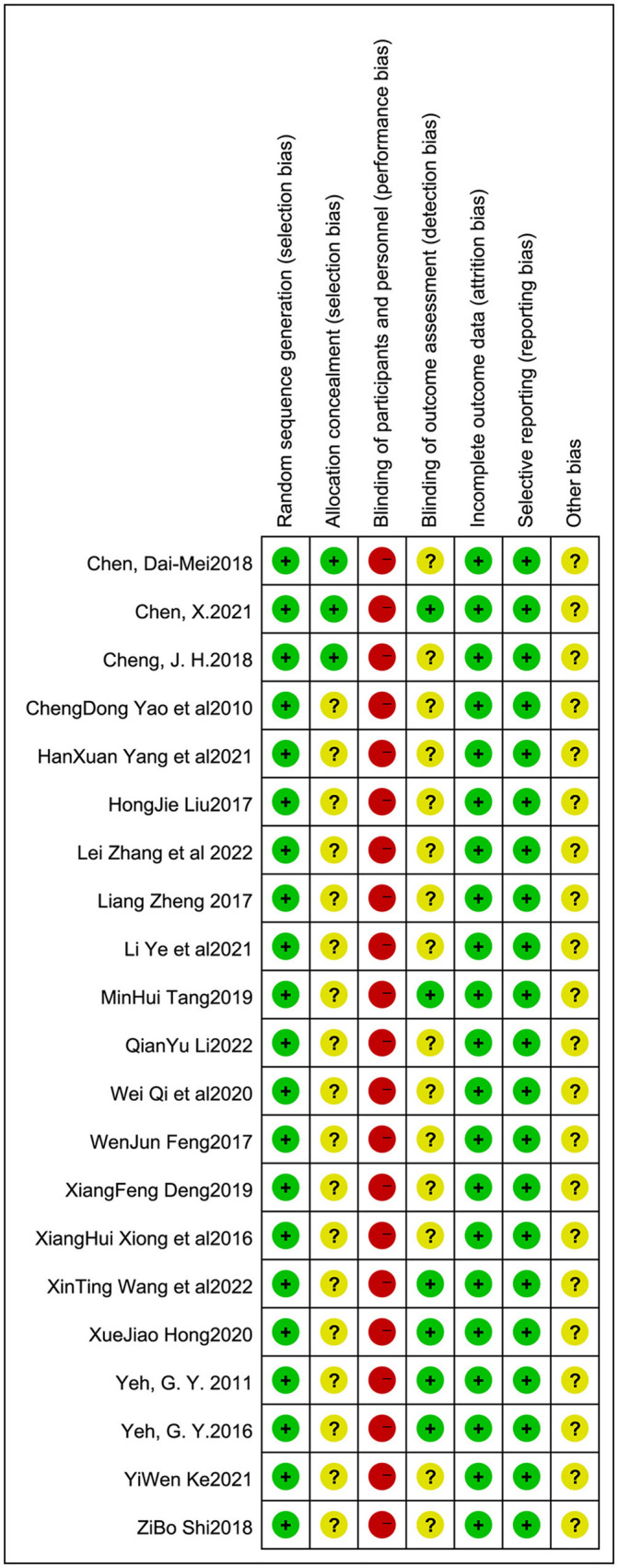
Bias risk summary: judgment of risk of bias and items with bias included in the studies; “+”, low risk; “–”, high risk; “?”, unclear.

The quality of the study was evaluated using Review Manager version 5.3. We assessed the risk of bias in all the included studies. All 21 (12–32) included studies mentioned “randomization,” of which four mentioned allocation concealment. Participants could not be blinded due to the intervention; therefore, all articles were at a high risk of blinding of participants and personnel. Six mentioned the blind method of outcome assessment. The risk of incomplete outcome data was low in all trials. All studies had an unclear risk of bias in selective reporting. The detailed results of the bias risk assessment are summarized in Figures 2, 3.

## 4. Meta-analysis results

### 4.1. Left ventricular ejection fraction

**Figure 4:**
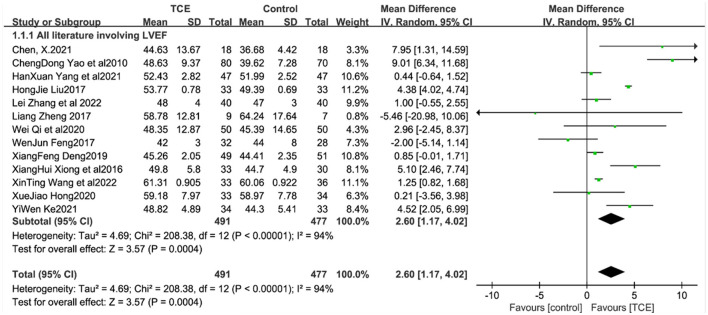
The effect of Traditional Chinese exercises on patients' LVEF.

LVEF values in patients with CHF were reported in 13 studies (12–22, 25, 26), which showed that LVEF values were higher in the trial group than in the control group. Meta-analysis showed a statistically significant difference in LVEF values in the trial group compared with the control group [MD = 2.60, 95% CI (1.17, 4.02), *P* < 0.001] due to high heterogeneity between studies (*I*^2^ = 94%, *P* < 0.001) (Figure 4).

**Figure 5:**
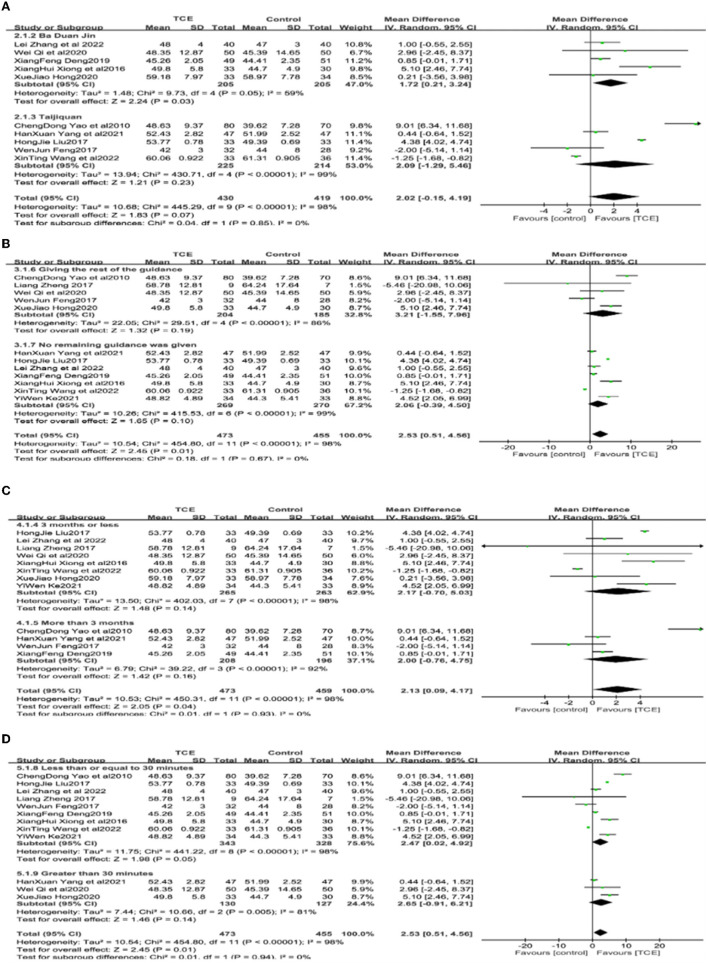
**(A–D)** Subgroup of the effect of Traditional Chinese exercises on patients' LVEF.

Because of the large bias in LVEF results across studies, subgroup analyses were performed with different types of gong methods, whether instructions other than drugs and gong methods were given, length of intervention (≤ 3; >3 months), and length of single intervention (≤ 30; >30 min) as subgroup variables (Figure 5).

In the subgroup analysis with an intervention using different types of exercises as subgroup variables, in which Bada Duan Jin and Taijiquan were used in five studies each [14–17, 19–21, 23, 27, respectively], the analysis was performed using a random effects model. The results showed that the LVEF values in the studies with Bada Duan Jin experimental group was statistically significant (*P* = 0.03) compared with the control group. However, in the studies in which the intervention exercises were combined using different gong methods, results showed that there was no statistically significant difference between the groups (*P* = 0.23) (Figure 5A).

In the subgroup analysis with the subgroup variable of whether or not to give instructions other than drugs and exercises, seven studies (13, 16, 17, 19, 21, 22) mentioned administering drugs and exercise interventions, while five studies (14, 18, 20, 25, 26) additional instructions were given to patients such as health education and emotional guidance (*P* = 0.19 and *P* = 0.10, respectively) (Figure 5B).

In a subgroup analysis with intervention duration as a subgroup variable, eight studies (13, 15, 17–20, 22, 25) with intervention duration ≤ 3 months and four studies (14, 16, 21, 26) with intervention duration >3 months were analyzed using a random effects model. The results showed that the LVEF values were not statistically significant in the experimental group compared to the intervention group for intervention lengths of <or > 3 months (*P* = 0.14, *P* = 0.16) (Figure 5C).

In a subgroup analysis with a single intervention duration as a subgroup variable, it was shown that nine studies (13–17, 19, 21, 22, 25, 26) had a single intervention duration of ≤ 30 min, and three studies (16, 18, 20) had a single intervention duration >30 min, which were analyzed using a random effects model. The results showed that the LVEF values were not statistically significant in the experimental group compared to the control group for single intervention durations of <or > 30 min (*P* = 0.05 and *P* = 0.14, respectively) (Figure 5D).

### 4.3. Maximum oxygen uptake (VO_2_max)

**Figure 6:**
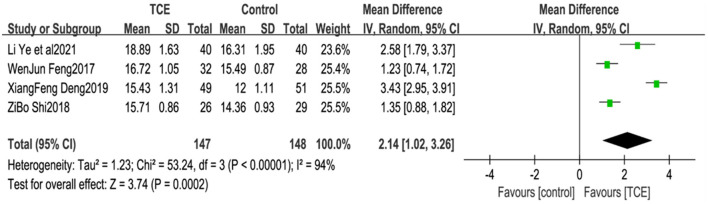
The effect of Traditional Chinese exercises on patients' VO2max.

Four studies (21, 26, 27, 29) reported patient VO_2_max, which is an indicator of aerobic exercise capacity in humans. Owing to the large heterogeneity of the included studies (*P* < 0.001, *I*^2^= 94%), meta-analysis using a random effects model showed that the VO_2_max levels in the group using TCEs were greater than those in the control group, and the difference between the two groups was statistically significant [MD = 2.14, 95% CI (1.02, 3.26), *P* < 0.001] (Figure 6).

### 4.4. Anaerobic threshold

**Figure 7:**
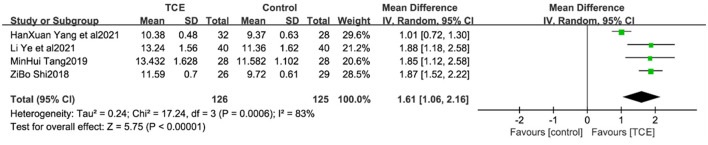
The effect of Traditional Chinese exercises on patients' AT.

Four studies (19, 24, 27, 29) reported patient AT levels, which are indicators of the ability to perform daily activities, and are closely related to cardiac function classification. Due to the large heterogeneity of the included studies (*P* < 0.001, *I*^2^ = 83%), a meta-analysis using a random-effects model showed that AT levels were greater in the TCE group than in the control group, and the difference between the two groups was statistically significant [MD = 1.61, 95% CI (1.06, 2.16), *P* < 0.001] (Figure 7).

### 4.5. Quality of life

**Figure 8:**
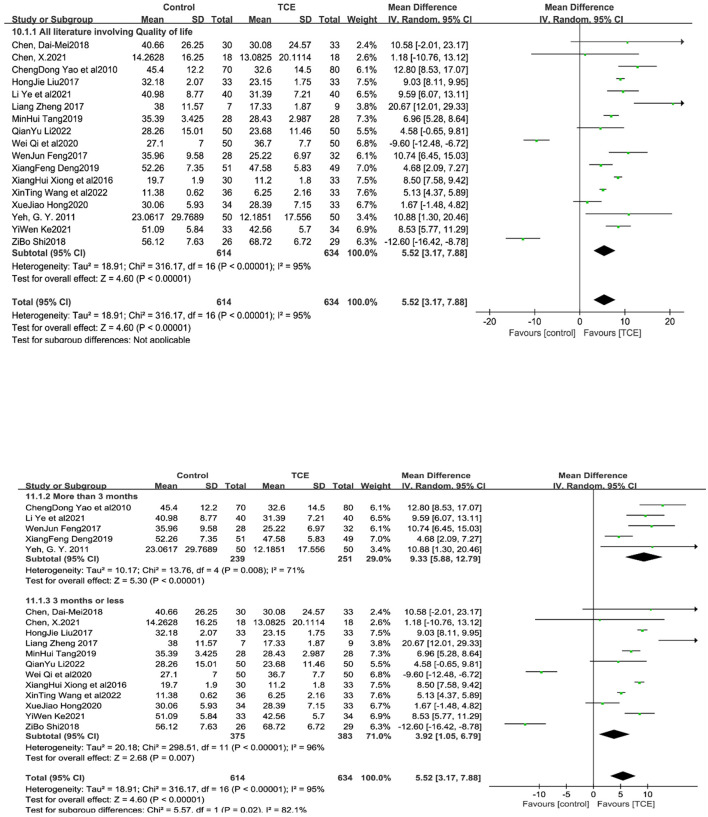
The effect of Traditional Chinese exercises on patients' quality of life (included subgroup analysis).

Seventeen studies (12–14, 17–27, 29, 30, 32) reported quality of life scores in patients with CHF. All the included study used the uniform Minnesota Malfunctional Heart Quality of Life Scale (MLHFQ) for scoring. Due to the high heterogeneity of the included studies (*I*^2^ = 95%, *P* < 0.001), a random-effects model was used for the analysis. The results of the meta-analysis showed that the test group had a statistically significant difference from the control group in terms of QOL scores [MD = 5.52, 95% CI (3.17, 7.88), *P* < 0.001]. Subgroup analyses were performed using the subgroup indicator of intervention duration (≤ 3 and >3 months). Of the subgroup analyses with intervention duration, five studies (14, 21, 26, 27, 30) had intervention duration >3 months, and 12 studies (12, 14, 17–20, 22–25, 29, 32) had intervention durations ≤ 3 months. The heterogeneity of the included studies was high and a meta-analysis with a random-effects model was used. The results showed that the quality of life was statistically significant in the experimental group of different time studies of intervention feats, compared to the intervention group (*P* < 0.001 and *P* = 0.007, respectively) (Figure 8).

### 4.6. 6MWD

**Figure 9:**
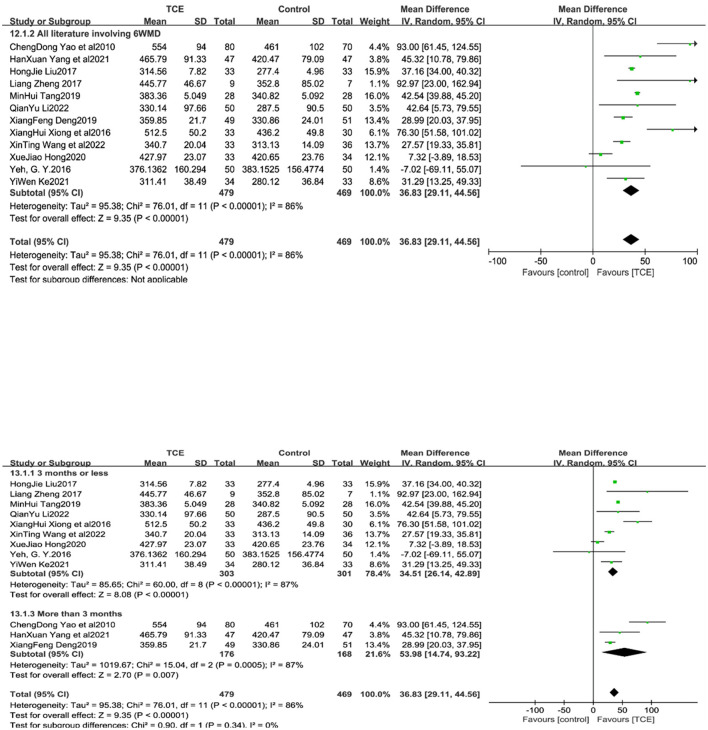
The effect of Traditional Chinese exercises on patients' 6MWD (included subgroup analysis).

Twelve studies (13, 14, 16–19, 21–24, 28) reported 6MWD in patients with CHF, and all 6MWD were higher in the trial group than in the control group. Due to the high heterogeneity of the included studies (*I*^2^ = 86%, *P* < 0.001), a random-effects model was used for the analysis. The results of the meta-analysis showed that the test group differed significantly from the control group in terms of QOL scores [MD = 36.83, 95% CI (29.11, 44.56), *P* < 0.001]. In the subgroup analysis of interventions by intervention duration, three studies (14, 16, 21) and (13, 17, 19, 22–25, 28) had an intervention duration greater than and ≤ 3 months, respectively. The heterogeneity of the included studies was high and a meta-analysis with a random-effects model was used. The results showed that the QOL was statistically significant in the experimental group of different time studies of intervention feats, compared to the intervention group (*P* < 0.001 and *P* = 0.007, respectively) (Figure 9).

### 4.7. Single-item TCM symptom scores in CHF patients

#### 4.7.1. Tiredness and fatigue

**Figure 10:**
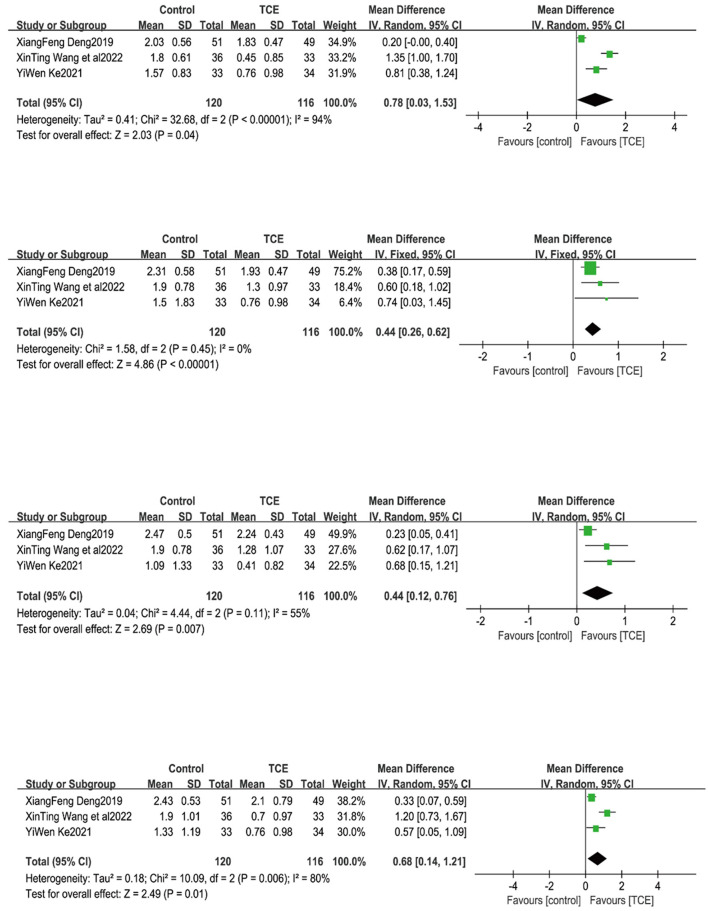
The effect of Traditional Chinese exercises on patients' single-item TCM symptom scores.

Three studies (17, 20, 22) reported the level of fatigue and weakness in the single-item TCM score of patients. Owing to the large heterogeneity of the included studies (*P* < 0.001, *I*^2^= 94%), meta-analysis using a random-effects model showed that the level of fatigue and weakness in the group using TCEs was better than that in the control group, and the difference was statistically significant when comparing the two groups [MD = 0.78, 95% CI (0.03, 1.53), *P* = 0.04] (Figure 10).

#### 4.7.2. Shortness of breath

Three studies (17, 20, 22) reported the level of shortness of breath in the single-item TCM score of patients. Due to the small heterogeneity of the included studies (*P* = 0.45, *I*^2^ = 0%), meta-analysis using a fixed-effects model showed that the level of shortness of breath in the group using TCEs was better than that in the control group, and the difference was statistically significant when comparing the two groups [MD = 0.44, 95% CI (0.26, 0.62), *P* < 0.0001] (Figure 10).

#### 4.7.3. Facial puffiness and limb swelling

Three studies (17, 20, 22) reported the level of facial puffiness and limb swelling in the single-item TCM score of patients. Owing to the large heterogeneity of the included studies (*P* = 0.11, *I*^2^ = 55%), meta-analysis using a random-effects model showed that the level of fatigue and weakness in the group using TCEs was better than that in the control group, and the difference was statistically significant when comparing the two groups [MD = 0.44, 95% CI (0.12, 0.76), *P* = 0.007] (Figure 10).

#### 4.7.4. Palpitations

Three studies (17, 20, 22) reported the level of palpitations in the single-item TCM score of patients. Owing to the large heterogeneity of the included studies (*P* = 0.006, *I*^2^ = 80%), meta-analysis using a random-effects model showed that the level of fatigue and weakness in the group using TCEs was better than that in the control group, and the difference was statistically significant when comparing the two groups [MD = 0.68, 95% CI (0.14, 1.21), *P* = 0.01] (Figure 10).

## 5. Publication bias and sensitivity analysis

Funnel plots were used for the analysis when the number of studies in the meta-analysis was ≥10. The results showed partial asymmetry, suggesting the possibility of a publication bias. Sensitivity analysis was performed by comparing the differences between the effect sizes obtained from the different combined models and comparing the changes in the total effect sizes after excluding each study individually.

## 6. Discussion

### 6.1. Improving cardiac function and prognosis

The LVEF is an index related to the myocardial contractility. The stronger the myocardial contractility, the more the stroke volume and the higher the LVEF. A meta-analysis showed that TCEs can effectively improve the LVEF of patients, which is consistent with a previous study by Ren et al. (33). The benefits of TCEs, such as Taijiquan, for patients with chronic heart failure have been included in relevant guidelines^2^. This is due to the fact that traditional exercises such as Taijiquan can make cardiomyocytes compensately thickened, resulting in the overall enhancement of myocardial contractility, so that the stroke volume and ejection fraction are correspondingly increased (34). In a subgroup analysis of LVEF, a possible source of heterogeneity was found in the study (15, 18–21), and the reason for this was that the TCEs used in the study were Ba Duan Jin, whereas the exercises used in the other included studies were Taijiquan, Yi Jin Jing, and Liu Zi Jue. The reasons for this need to be discussed further. In terms of improving myocardial contractility in heart failure patients, the use of Bada Duan Jin may be superior to Taijiquan, and more clinical studies are needed to prove the specific mechanism. Ba Duan-jin emphasized keeping gentle and steady lower limb movements, which increased the endurance of lower limb skeletal muscles, enhanced the strength and strength of muscle fibers, improved muscle perfusion and metabolism, and increased the total volume density of mitochondria and the capacity density of cytochrome c oxidase in muscle fiber cells (35, 36). In future clinical practice, healthcare providers can carry out training of TCEs to improve myocardial contractility during the rehabilitation phase according to the condition of heart failure patients. However, owing to the small sample size and heterogeneity of the studies included in this analysis, more clinical trials are needed to corroborate the results in the future.

### 6.2. Enhancement of exercise capacity

Meta-analysis results showed that TCM methods could effectively enhance patients' maximal oxygen consumption, anaerobic threshold level, and 6MWD, both of which represented an increase in patients' exercise endurance and intensity. This may be because abdominal breathing in some movements of gong methods such as Yi Jin Jing and Taijiquan can regulate parasympathetic activity and heart rate variability, thus improving cardiopulmonary function (37). At the same time, abdominal breathing will increase the range of diaphragm movement, increase the maximum ventilation and reduce the residual volume, improve the hypoxia state during exercise and increase exercise endurance (38). Of interest is that in the subgroup analysis of 6MWD, the results showed that those with ≤ 3 months of intervention were more statistically significant compared to those with >3 months of intervention. However, since there were only three studies with an intervention period >3 months, there may be a bias in the results, and more long-period intervention trial studies are needed to support this result in the future.

### 6.3. Reducing symptom clusters and improving QOL

A previous study Huang et al. (39) has shown that patients' symptoms and quality of life are closely related. Meta-analysis results showed that fatigue, shortness of breath, swelling of the face and limbs, and palpitations improved in the individual TCM symptom scores of patients; however, since only three studies were included for each outcome, there may be false negatives. TCEs can stimulate yang, regulate qi and blood, regulate vital qi of human meridians and viscera, reduce heart burden, improve the ability of transporting and utilizing oxygen in human blood circulation, thus reducing oxygen consumption of myocardium, alleviating dyspnea symptoms of heart failure and improving quality of life (40). TCEs like Tai Ji can effectively promote the excitement of the right hemisphere, inhibit the activity of the left hemisphere, increase the α brain waves, endorphins and catecholamines of practitioners, make practitioners feel pleasant and enhance their quality of life (41, 42). The results suggest that TCEs can be effective in improving the QOL, and the timing of the intervention may be a source of heterogeneity; however, further discussion is needed.

## 7. Limitations of the study

Slight differences in the intervention protocols and sample population characteristics due to the included studies may lead to increased heterogeneity of the results. In the case of physiological index measurements, the use of different instruments may lead to differences in the results and bias in measurement. In terms of QOL, single TCM symptom scores and subjective research instruments were mostly used, and the lack of uniform criteria may have affected the reliability of the results. In addition, flaws in the included studies, such as the inability to perform hidden allocation, failure to elaborate on whether measurement bias was performed, and follow-up bias may have affected the reliability of the results.

## 8. Summary

The present study clarified the beneficial effects of subjecting patients with CHF to TCEs on their recovery, mainly by enhancing LVEF, VO_2_max, anaerobic threshold, quality of life, and single-item TCM scores (fatigue, shortness of breath, floating limbs, and palpitations) in patients with CHF. However, the source of heterogeneity in the quality of life is unclear. Future research can be compared according to patients' different cardiac function grades, so as to provide more specific exercise recommendations for patients with heart failure. Also more high-quality, large-sample RCTs are needed to specifically analyse the above outcome indicators for further quantitative clinical promotion of TCEs in the rehabilitation of patients with CHF.

## Data availability statement

The original contributions presented in the study are included in the article/Supplementary material, further inquiries can be directed to the corresponding author.

## Author contributions

MD and ZL: conceptualization and methodology. MD: data curation and analysis and original writing. SH and HC: review and editing. JY, DG, and WL: data curation and validation. All authors have made substantive contributions to this study in regard to design and implementation, read, and approved the final manuscript.

## Footnotes

^1^Available online at: https://www.researchgate.net/publication/358352812_Guidelines_for_Cardiac_Rehabilitation_Programs_AACVPR.

^2^Available online at: https://www.moh.gov.my/moh/resources/penerbitan/CPG/CPG%20Heart%20Failure%202019.pdf.
