# Supplementary material for: Effects of traditional Chinese exercises on the rehabilitation of patients with chronic heart failure: A meta-analysis
Source: Front Public Health. 2023 Feb 23;11:1139483. doi: 10.3389/fpubh.2023.1139483 (PMC9995941; doi:10.3389/fpubh.2023.1139483)

**Appendix：**

1. Search strategies: Ten databases in Chinese and English language were searched, including:

China National Knowledge Infrastructure (CNKI), WeiPu, Wanfang, China Biology Medicine disc, National Medical Journal of China, PubMed, Cochrane Library, Web of Science, EBSCO, Embase database. Since search strategies were the same for Chinese database, only search criteria for the representative PubMed and CNKI were listed as follows.

The search model was as follows:

PubMed 2022/11/25

#1 ("Qigong"[Mesh]) OR ("Tai Ji"[Mesh])

#2 (Liuzijue) OR (Six Letters Formula) OR (Six Character Formula) OR (Six-Character Pithy) OR (yijinjing) OR (Fitness Qigong Yijinjing) OR (wuqinxi) OR (five animal exercise) OR (five mimic animal exercise) OR (five arumal Exercise) OR (baduanjin) OR (eight trigrams boxing) OR (eight-section brocade) OR (eight pieces brocade) OR (wuxingzhang) OR (zhanzhuanggong) OR (standing qigong) OR (Zhan zhuang posture) OR (huichungong)

#3 ("Heart Failure"[Mesh])

#4 #1 OR #2

#5 #4 AND #3

CNKI 2022/11/25

#1KY= ('baduanjin'+'TaiJi'+'wuqinxi'+'Liuzijue'+'Qigong'+'yijinjing'+'wuxingzhang'+' zhanzhuanggong' +'huichungong')

#2 KY=('Chronic heart failure' + 'heart failure' + 'cardiac insufficiency'）

#3 #1 AND #2

1. Basic characteristics of the included literature


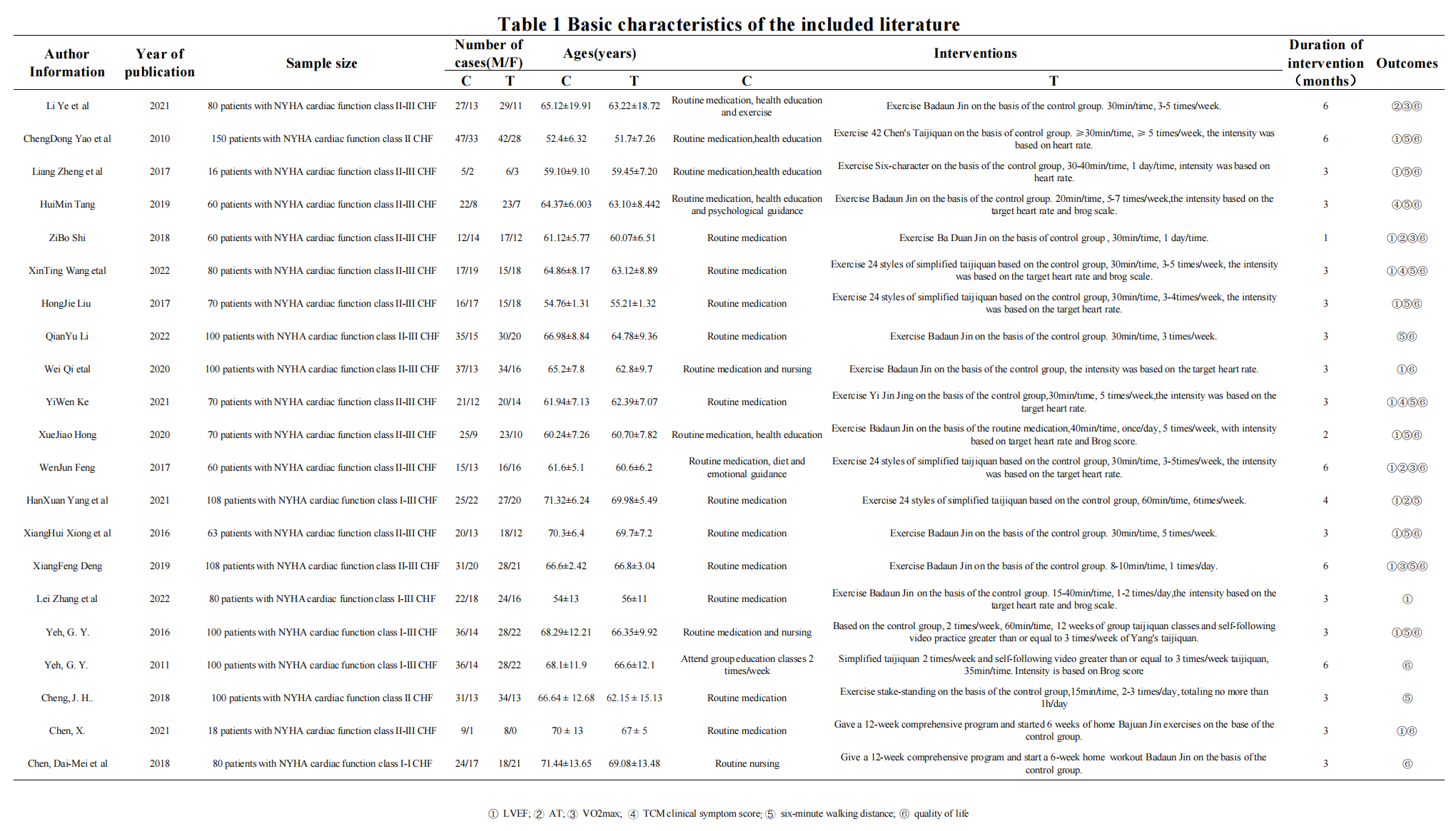


1. Sensitivity analysis
   1. Left ventricular ejection fraction


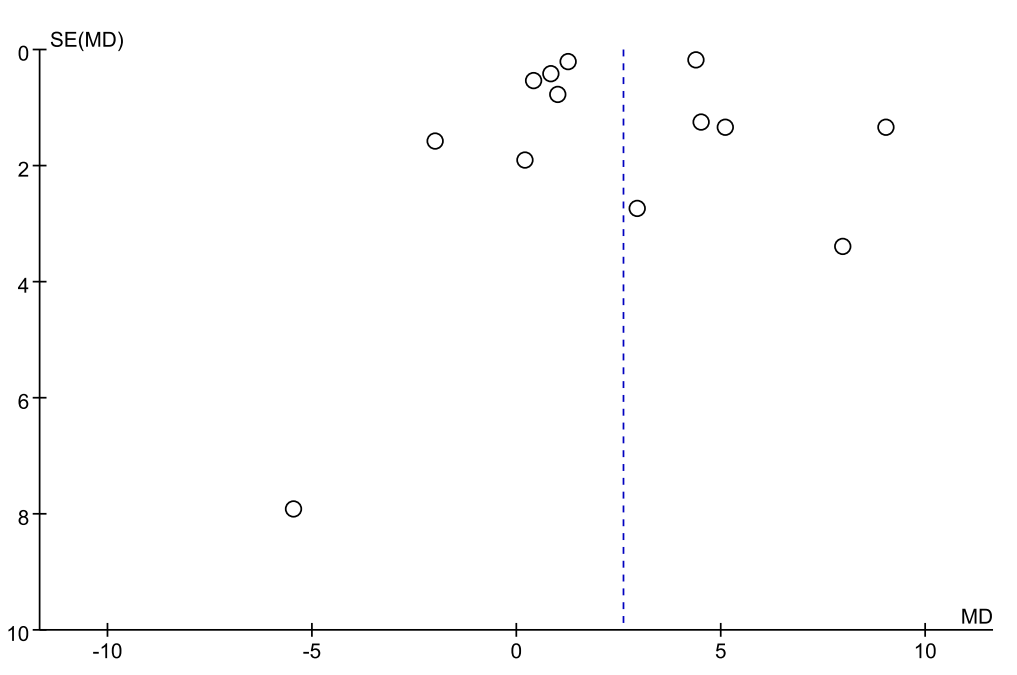


- 1. ~~N-terminal B-type natriuretic peptide~~
  2. Maximum oxygen uptake


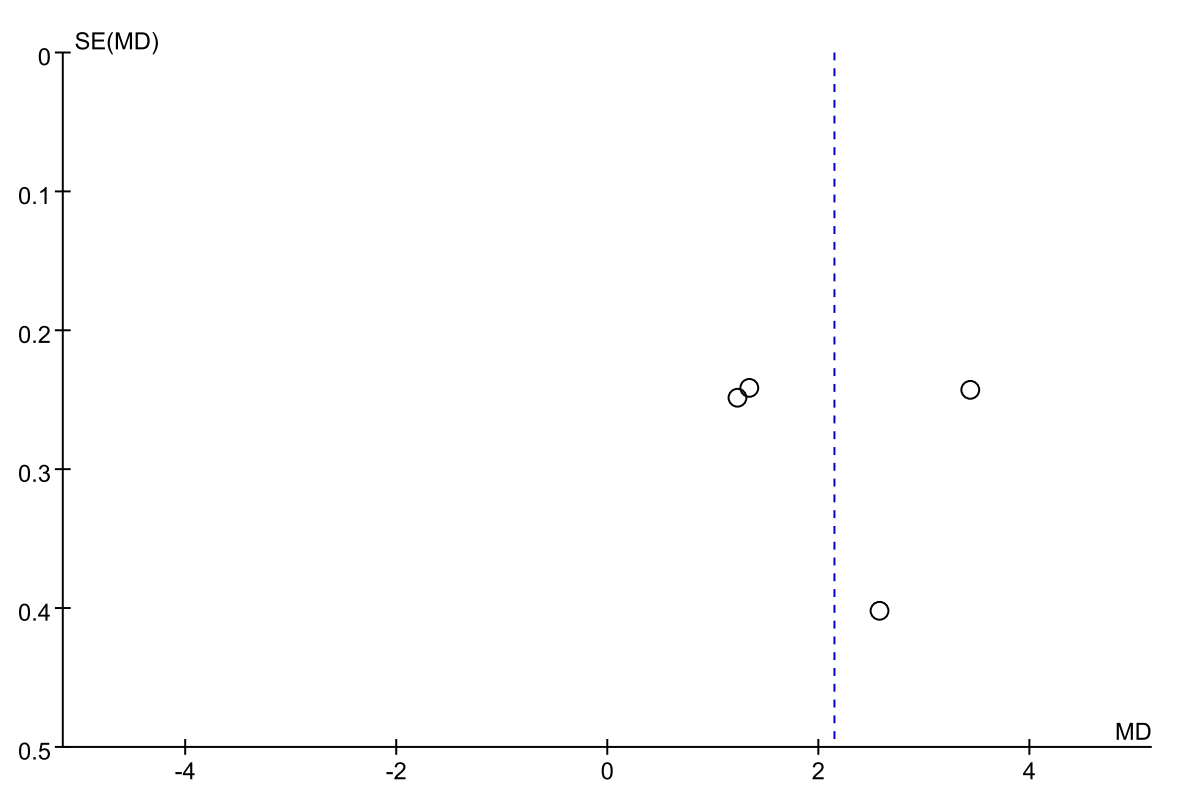


- 1. Anaerobic valve


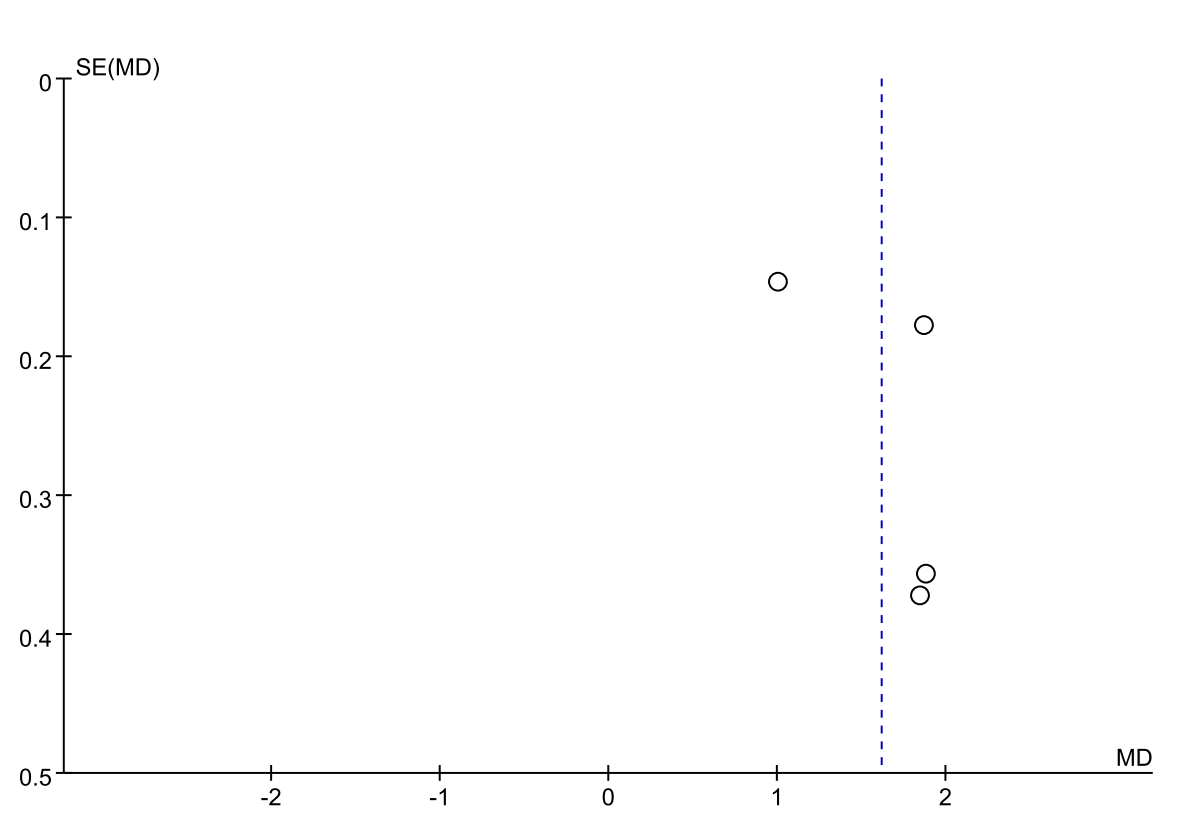


- 1. Quality of life


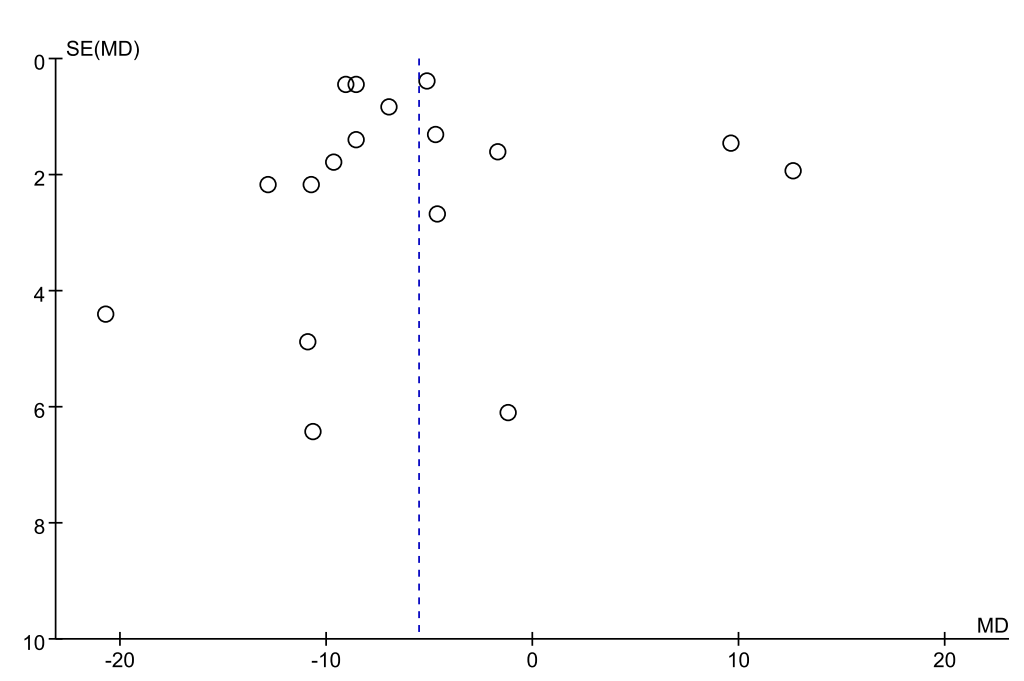


- 1. 6MWD


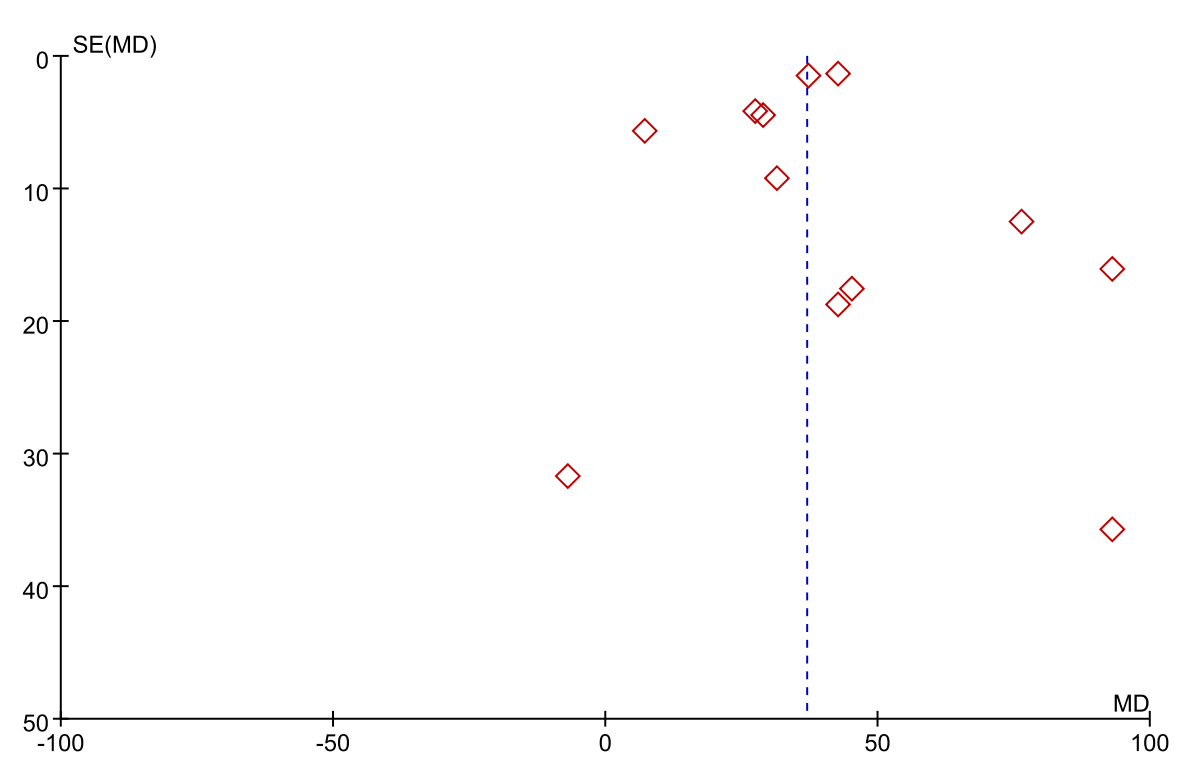


- 1. Single-item TCM symptom scores in CHF patients

3.7.1 Tiredness and fatigue


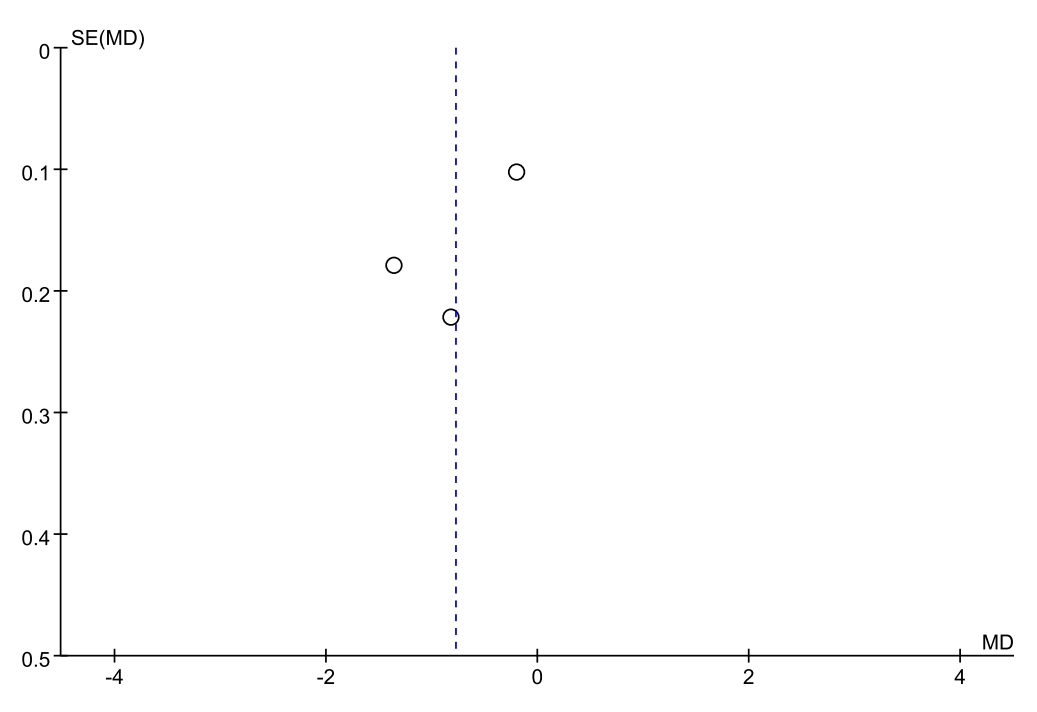


3.7.2 Shortness of breath


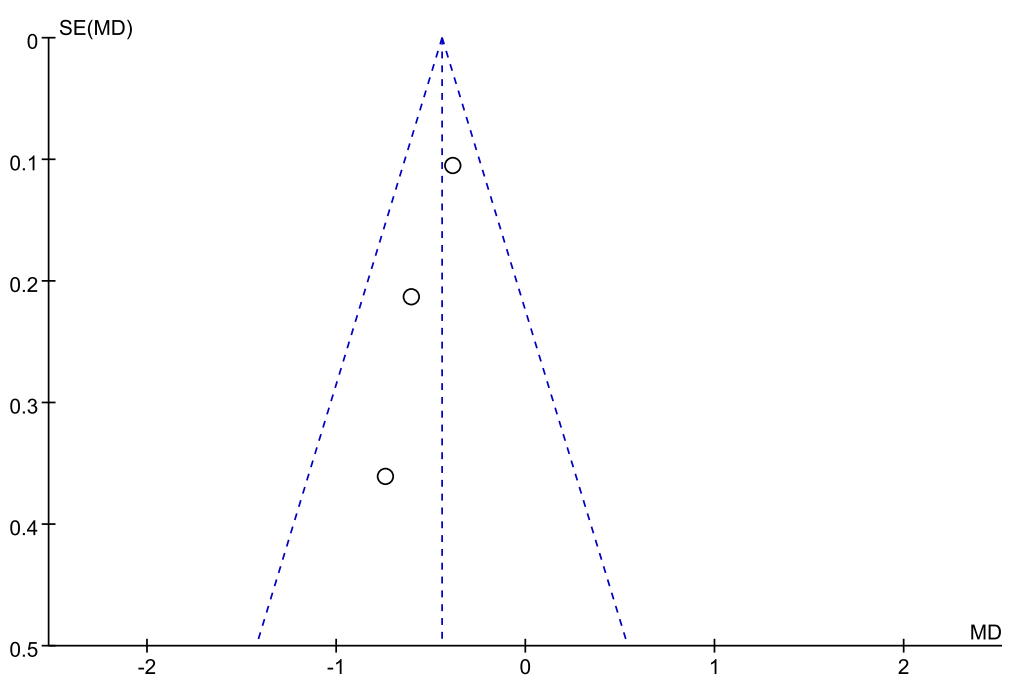


3.7.3 Facial puffiness and limb swelling


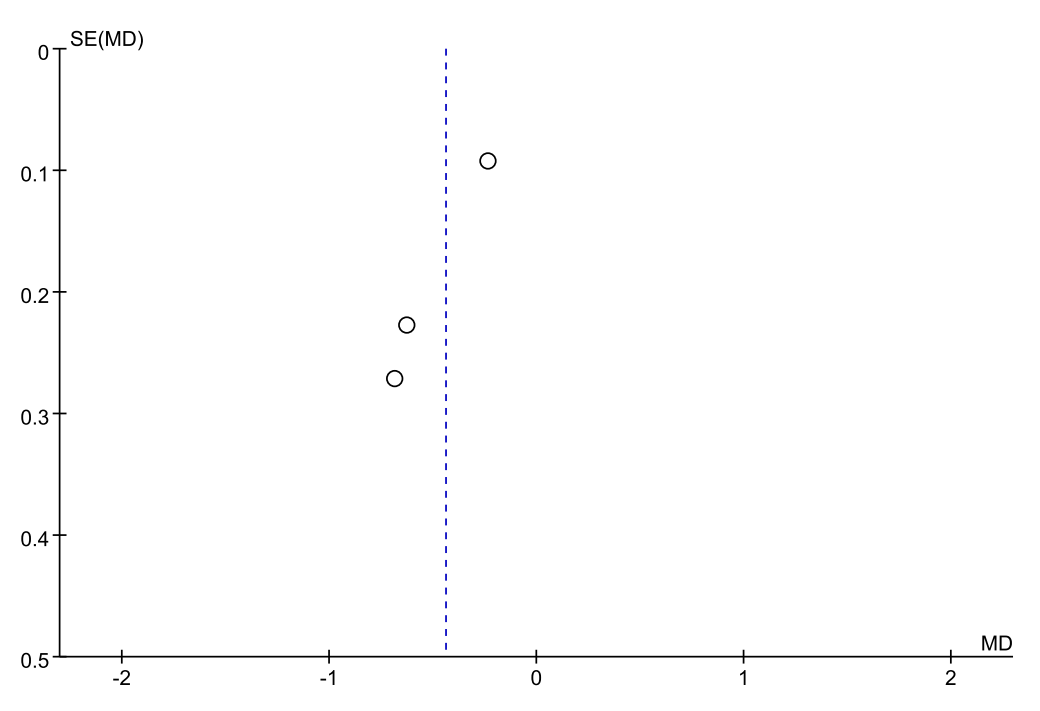


3.7.4 Palpitations


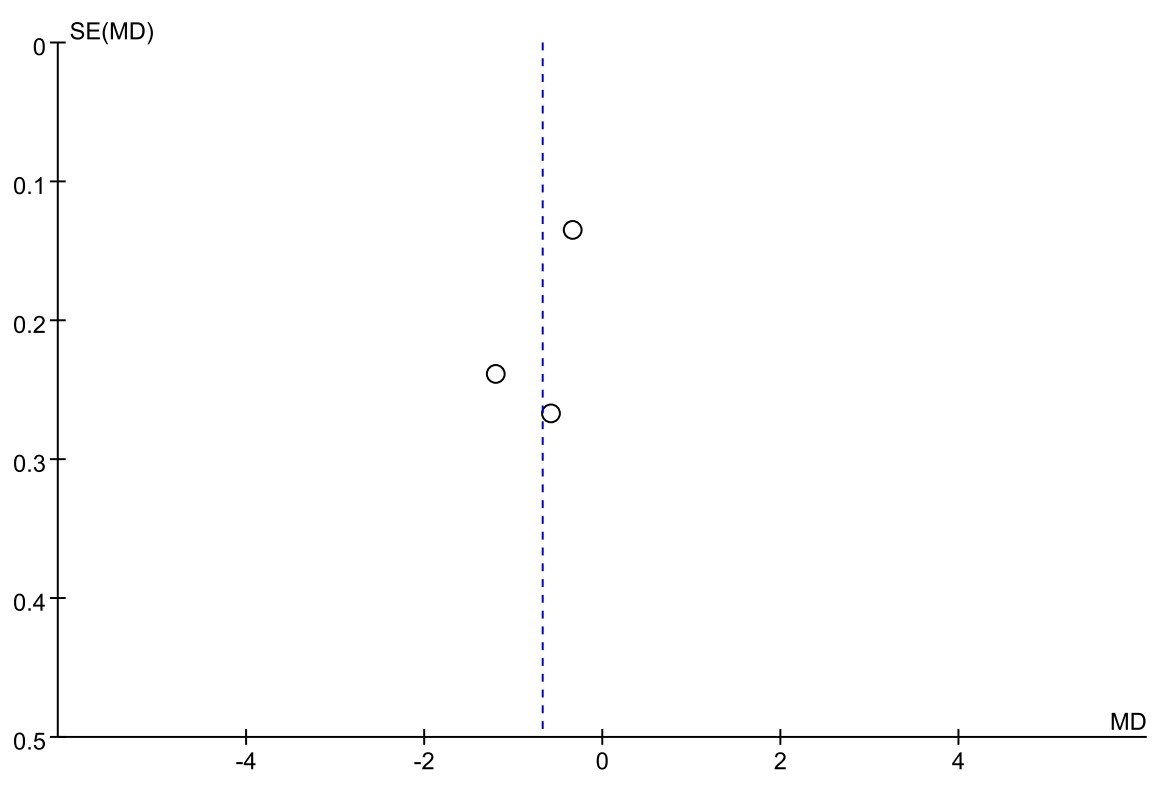

Supplement: Supplementary file 1 [file Data_Sheet_1.doc]
